# Relationship between the Inflammatory Molecular Profile of Breast Carcinomas and Distant Metastasis Development

**DOI:** 10.1371/journal.pone.0049047

**Published:** 2012-11-08

**Authors:** Noemí Eiró, Lucía González, Luis O. González, Belen Fernandez-Garcia, Maria Luz Lamelas, Laura Marín, Salomé González-Reyes, José Manuel del Casar, Francisco J. Vizoso

**Affiliations:** 1 Unidad de Investigación, Fundación Hospital de Jove, Gijón, Asturias, Spain; 2 Servicio de Anatomía Patológica, Fundación Hospital de Jove, Gijón, Asturias, Spain; 3 Servicio de Ginecología, Fundación Hospital de Jove, Gijón, Asturias, Spain; 4 Servicio de Cirugía General, Fundación Hospital de Jove, Gijón, Asturias, Spain; Institut de Génomique Fonctionnelle de Lyon, France

## Abstract

Inflammatory conditions may promote tumor progression and aggressiveness. In previous reports, we found a group of breast cancer tumors characterized by metalloprotease-11 (MMP-11) expression by intratumoral mononuclear inflammatory cells (MICs), which was associated with distant metastasis development. Thus, in the present study we evaluated the relationship between MMP-11 expression by MICs, distant metastasis development, and a wide panel of inflammatory factors in breast carcinoma. In an initial approach, we analyzed 65 factors associated with tumor progression and inflammation, in a tumor population classified in good or bad prognosis, based on MMP-11 expression by intratumoral MICs. The most differentially expressed factors were then analyzed in a wider tumor population classified according to MMP-11 expression by MICs and also according to metastasis development. These analyses were carried out by Real-time PCR. The results showed that of the 65 starting factors analyzed, those related with MMP-11 expression by MICs were: IL-1, −5, −6, −8, −17, −18, MMP-1, TIMP-1, ADAM-8, −10, −15, −23, ADAMTS-1, −2, −15, Annexin A2, IFNβ, Claudin-3, CCL-3, MyD88, IRAK-4 and NFκB. Of them, factors more differentially expressed between both groups of tumors were IL-1, IL-5, IL-6, IL-17, IFNβ and NFκB. Thereafter, we confirmed in the wider tumor population, that there is a higher expression of those factors in tumors infiltrated by MMP-11 positive MICs. Altogether these results indicate that tumors developing worse prognosis and identified by MMP-11 expression by intratumoral MICs, shows an up-regulation of inflammatory-related genes.

## Introduction

Inflammatory cells and immune mediators in tumor microenvironment influence tumor progression and metastasis in cancers, such as breast cancer [1]. Historically, tumor-infiltrating leukocytes have been considered as an intrinsic defense mechanism against tumor development. However, increasing evidences indicate that leukocyte infiltration may favor tumor development by promoting angiogenesis, growth, and invasion. This may happen because inflammatory cells influence cancer promotion by secreting cytokines, growth factors, chemokines and proteases, which stimulate proliferation and invasiveness of cancer cells. Consequently, events and molecules implicated in this cross-talk between tumor and inflammatory microenvironment may emerge as attractive targets in anticancer intervention with significant clinical impact.

In previous reports, we found that 32% of breast carcinomas analyzed contained mononuclear inflammatory cells (MICs) in the intratumoral stroma with a high metalloproteases and tissue inhibitor metalloproteases (MMPs/TIMPs) expression profile, which is associated with a higher rate of distant metastasis development (97.6%), as compared with patients whose MICs had a low MMPs/TIMPs expression profile and are associated with a lower rate of distant metastasis (26.9%). Those prometastatic-related MICs were characterized by overexpression of MMP-7, 9, 11, 13 and 14, and TIMP-1 and 2 [2], [3]. Of them, stromelysin-3 (MMP-11) was the most frequently expressed in this MICs population (85.7% *vs.* 4.6% in the low MMPs/TIMPs profile group), and therefore the expression of this factor is considered as a useful biological marker of these prometastatic-related MICs.

The high occurrence of distant metastasis in patients whose tumors are infiltrated by MICs overexpressing MMPs and TIMPs may be, in part, because MMPs play an essential role in the degradation of the stromal connective tissue and basement membrane components, which are key elements in tumor invasion and metastasis. MMPs are also able to impact on tumor cell behavior *in vivo* because of their capacity to cleave growth factors, cell surface receptors, cell adhesion molecules, and chemokines/cytokines [4], [5], [6]. Furthermore, by cleaving proapoptotic factors, MMPs induce a more aggressive phenotype as a consequence of generation of apoptotic resistant cells [4]. MMPs also regulate cancer-related angiogenesis, both positively through their ability to mobilize or activate proangiogenic factors [7], or negatively via generation of angiogenesis inhibitors, such as angiostatin and endostatin, cleaved from large protein precursors [8]. Moreover, it is now accepted that TIMPs are multifactorial proteins involved in the induction of proliferation and the inhibition of apoptosis [9], [10].

Nevertheless, we consider that these tumors containing MMPs/TIMPs-overexpressing MICs, and with a higher rate of distant metastasis, may also express other inflammatory factors, which may be potential biological markers of tumor aggressiveness and/or therapeutic targets in breast cancer. Therefore, the aim of the present study was to evaluate the relationship between MMP-11 expression by intratumoral MICs, distant metastasis development, and a wide panel of biological parameters related to tumor progression and inflammation in breast carcinoma.

## Materials and Methods

### Ethics Statement

Women were treated according to the guidelines used in our Institution. Written informed consent, approved by our Institution’s Ethics and Investigation Committee, was obtained from all patients before the evaluation of tumor samples. The study adhered to National regulations and was approved by our Institution’s Ethics and Investigation Committee.

### Patient Selection and Study Design

We selected women with the following inclusion criteria: early invasive breast cancer (without distant metastasis at initial diagnosis), at least 6 histopathologically assessed axillary lymph nodes, T1 or T2 size tumors and a minimum of 10 years of follow-up for women without tumor recurrence. The exclusion criteria were the following: metastatic disease at presentation, prior history of any kind of malignant tumor, bilateral breast cancer at presentation, having received any type of neoadjuvant therapy, development of locoregional recurrence during the follow-up period and development of a second primary cancer (distant recurrence cases were not excluded).

From 320 patients fulfilling these criteria, diagnosed and treated between 1990 and 2005, we selected 6 patients whose tumors had MMP-11 negative MICs (Group A) and 6 patients whose tumors had MMP-11 positive MICs (Group B). In both groups of tumors we analyzed the expression of 65 factors (Table 1) by PCR. Proteins showing more significant differences between groups were then analyzed in a wider tumor population corresponding to 3 groups of patients, selected from the remainder patients who fulfilled the inclusion criteria, and stratified as follows: Group A1, 15 patients with MMP-11 negative intratumoral MICs and without distant metastasis during the follow-up period; Group A2, 15 patients with MMP-11 negative intratumoral MICs and with distant metastasis during the follow-up period; and Group B1, 15 patients with MMP-11 positive intratumoral MICs and with distant metastasis during the follow-up period. Given the number of breast cancers that meet the inclusion criteria, 15 patients per group is a representative sample of the population. It is important to emphasize that we did not find a critical number of patients to gather a group B2 (MMP-11 positive intratumoral MICs and without distant metastasis). Protein expression of the factors analyzed in the last three groups was confirmed by immunohistochemistry.

**Table 1:** Factors analyzed by real-time PCR.

Symbol	Official name	Symbol	Official name
**ADAM8**	A desintegrin and metalloprotease 8	**IL8**	Interleukin 8
**ADAM9**	A desintegrin and metalloprotease 9	**IL10**	Interleukin 10
**ADAM10**	A desintegrin and metalloprotease 10	**IL12**	Interleukin 12
**ADAM12**	A desintegrin and metalloprotease 12	**IL17**	Interleukin 17
**ADAM15**	A desintegrin and metalloprotease 15	**IL18**	Interleukin 18
**ADAM17**	A desintegrin and metalloprotease 17	**IRAK4**	Interleukin-1 receptor-associated kinase 4
**ADAM23**	A desintegrin and metalloprotease 23	**IRF3**	Interferon regulatory factor 3
**ADAM33**	A desintegrin and metalloprotease 33	**MMP1**	Matrix metalloprotease 1(interstitial collagenase)
**ADAMTS1**	ADAM metalloprotease with thrombospondin type 1 motif, 1	**MMP2**	Matrix metalloprotease 2 (gelatinase A)
**ADAMTS2**	ADAM metalloprotease with thrombospondin type 1 motif, 2	**MMP3**	Matrix metalloprotease 3 (stromelysin 1)
**ADAMTS4**	ADAM metalloprotease with thrombospondin type 1 motif, 4	**MMP7**	Matrix metalloprotease 7 (matrilysin)
**ADAMTS5**	ADAM metalloprotease with thrombospondin type 1 motif, 5	**MMP9**	Matrix metalloprotease 9 (gelatinase B)
**ADAMTS15**	ADAM metalloprotease with thrombospondin type 1 motif, 15	**MMP11**	Matrix metalloprotease 11 (stromelysin 3)
**ANGPT1**	Angiopoietin 1	**MMP13**	Matrix metalloprotease 13 (collagenase 3)
**ANGPT2**	Angiopoietin 2	**MMP14**	Matrix metalloprotease 14 (membrane-inserted)
**ANXA2**	Annexin A2	**MyD88**	Myeloid differentiation primary response gene 88
**CCL3**	Chemokine (C-C motif) ligand 3	**NFκB**	Nuclear factor kappa B
**CLDN3**	Claudin-3	**TEK**	TEK tyrosine kinase, endothelial
**COX2**	Cyclooxygenase 2	**TGFβ1**	Transforming growth factor beta 1
**CXCL16**	Chemokine (C-X-C motif) ligand 16	**TIMP1**	TIMP metalloprotease inhibitor 1
**CXCR4**	Chemokine (C-X-C motif) receptor 4	**TIMP2**	TIMP metalloprotease inhibitor 2
**EGF**	Epidermal growth factor	**TIMP3**	TIMP metalloprotease inhibitor 3
**FGF-2**	Fibroblast growth factor 2	**TLR2**	Toll-like receptor 2
**FN**	Fibronectin	**TLR3**	Toll-like receptor 3
**ICAM1**	Intercellular adhesion molecule 1	**TLR4**	Toll-like receptor 4
**IFNα**	Interferon alpha	**TLR5**	Toll-like receptor 5
**IFNβ**	Interferon beta	**TLR7**	Toll-like receptor 7
**IGF-1**	Insulin-like growth factor 1 (Somatomedin C)	**TLR9**	Toll-like receptor 9
**IGFBP-2**	Insulin-like growth factor binding protein 2	**TNF**	Tumor necrosis factor
**IL1**	Interleukin 1	**VEGFA**	Vascular endothelial growth factor A
**IL4**	Interleukin 4	**VEGFC**	Vascular endothelial growth factor C
**IL5**	Interleukin 5	**VEGFD**	Vascular endothelial growth factor D
**IL6**	Interleukin 6		

Patient characteristics are listed in Table 2. *Menopausal status was defined* as “postmenopausal” if 1 year was elapsed since the last menstrual period. For reporting the Histological Grade we used the Nottingham combined histologic grade (Elston-Ellis modification of Scarff-Bloom-Richardson grading system) [11]. For estrogen (ER) and progesterone (PgR) receptors evaluation we used mouse anti-ER (clone 1D5) diluted 1/50, and anti-PgR (clone PgR 636) diluted 1/50 (Dako, Glostrup, Denmark). Staining for ERs and PgRs was scored according to the method described by Allred et al. [12].

**Table 2:** Patient and tumor characteristics.

Characteristics	Group A1 N° (%)	Group A2 N° (%)	Group B1 N° (%)
**Total cases**	**15 (100)**	**15 (100)**	**15 (100)**
**Menopausal status**			
Premenopausal	4 (26.7)	5 (33.3)	5 (33.3)
Postmenopausal	11 (73.3)	10 (66.7)	10 (66.7)
**Tumoral size**			
T1	7 (46.7)	6 (40)	6 (40)
T2	8 (53.3)	9 (60)	9 (60)
**Nodal status**			
N(−)	5 (33.3)	6 (40)	7 (46.7)
N(+)	10 (66.7)	9 (60)	8 (53.3)
**Histological grade**			
Well Dif. (I)	5 (33.3)	4 (26.7)	5 (33.3)
Mod. Dif. (II)	7 (46.7)	7 (46.7)	5 (33.3)
Poorly Dif. (III)	3 (20)	4 (26.7)	5 (33.3)
**Estrogen receptors**			
Negative	6 (40)	9 (60)	8 (53.3)
Positive	9 (60)	6 (40)	7 (46.7)
**Progesterone receptors**			
Negative	5 (33.3)	11 (73.3)	9 (60)
Positive	10 (66.7)	4 (26.7)	6 (40)
**Adjuvant radiotherapy**			
No	6 (40)	8 (53.3)	9 (60)
Yes	9 (60)	7 (46.7)	6 (40)
**Adjuvant systemic therapy**			
Chemotherapy	6 (40)	8 (53.3)	7 (46.7)
Adjuvant Tamoxifen	2 (13.3)	2 (13.3)	4 (26.7)
Chemotherapy *plus* sequential Tamoxifen	5 (33.3)	3 (20)	2 (13.3)
No treatment	2 (13.3)	2 (13.3)	2 (13.3)

We evaluated 30 patients with tumors showing MMP-11 negative expression by MICs, without (Group A1) or with (Group A2) distant metastasis, and 15 patients with tumors showing MMP-11 positive expression by MICs and with distant metastasis (Group B1).

The endpoint of the study was distant metastatic relapse. The median follow-up period in patients without metastases was 85 months, and 46 months in patients who developed metastases.

### Tumor Tissue Handling and Immunohistochemistry

Breast carcinoma tissue samples were obtained at the time of surgery, routinely fixed (in 10% buffered formalin), paraffin-embedded and stored in our pathology laboratory. Serial 3 µm sections of these tumor samples were cut using a microtome (Leica Microsystems) and transferred to an adhesive-coated slide.

Immunohistochemistry was performed on tissue sections using a TechMate TM50 autostainer (Dako). To enhance antigen retrieval, tissue sections were treated in a PT-Link® (Dako) at 97°C for 20 min, in citrate buffer pH 6.1 for IL-1, −5, −6, −17, and in Tris-EDTA buffer pH 9 for IFNβ and NFκB, and then washed in phosphate buffered saline (PBS). Antibody for MMP-11 did not require antigen. The dilution for each antibody was as follows: 1/50 for IL-5; 1/200 for MMP-11; 1/300 for IL-17; 1/400 for IL-1, -6 and IFNβ; and 1/600 for NFκB. The negative control was DakoCytomation mouse or rabbit serum diluted at the same concentration as the primary antibody. Dilutions were made in antibody diluent (Dako) and incubated for 30 min to 2 h at room temperature. Breast tumor samples in which MMP-11 expression was confirmed by Western-blot analysis, were used as positive controls, as shown previously [2], [13]. Endogenous peroxidase activity was blocked by incubating the slides in peroxidase blocking solution (Dako) for 5 min. The EnVision Detection kit (Dako) was used as the detection system. Sections were counterstained with haematoxylin, dehydrated with ethanol and permanently coverslipped.

In the present work we evaluated the immunoreactivity on stromal MICs exclusively. Each evaluated field (400× power objective) contained at least 10 stromal MICs. We considered a positive immunostaining, for MMP-11 by MICs, when at least 10% of MICs showed a positive immunostaining at each evaluated field in every case, as established previously [2]. We used several markers to distinguish mononuclear inflammatory cells: CD3 for T-cells, CD20 for B-cells and CD68 for macrophages, all from Dako. Ten fields per case, corresponding to areas with higher immunostaining and without necrosis, were evaluated for CD3, CD20 and CD68 cell counting in a 1 mm^2^ final area.

We also evaluated the immunohistochemical staining for IL-1, −5, −6, −17, IFNβ and NFκB by each main cellular type: tumor cells, fibroblast and MICs. Stromal cells were distinguished from cancer cells because the latter are larger in size, and fibroblasts since they are spindle-shaped whereas mononuclear inflammatory cells are small round cells. Moreover, while cancer cells are arranged forming either acinar or trabecular patterns, stromal cells are spread, and also we used several markers to distinguish mononuclear inflammatory cells as describe above. We considered a positive immunostaining, for these inflammatory factors by any of these cell types, when at least 10% of cells showed a positive immunostaining at each evaluated field in every case.

### Real-time PCR

Total RNA was isolated from formalin-fixed paraffin-embedded tissue blocks using the Nucleospin® FFPE RNA Kit (Macherey-Nagel), including DNase treatment. We assessed the quality and quantity of extracted RNA using agarose gel electrophoresis and optical density measurements (NanoDrop Technologies, Wilmington, US). First strand cDNA was synthesized using the High Capacity cDNA Reverse Transcription kit (Applied Biosystems) following the manufacturer’s instructions. Reverse transcription step was carried out using the following program: 25°C for 10 min, 37°C for 120 min and 85°C for 5 sec. Expression of the different factors and β-actin (internal control) were assessed by real-time PCR using Fast SYBR Green Master Mix (Applied Biosystems) in an ABI Prism 7900 HT thermocycler (Applied Biosystems) with the following cycling conditions: 95°C for 20 sec, 40 cycles of 95°C for 1 sec and 60°C for 20 sec. Primers used are listed in Table 3.

**Table 3:** Primers sequences used for real-time PCR analysis (listed 5′- to -3′ end).

Symbol	Primers sequences	Symbol	Primers sequences
**ADAM8**	F- GTGAATCACGTGGACAAGCTAT	**IL8**	F- TCTCAGCCCTCTTCAAAAACTTCTC
	R- TTCTTGCTGTGGTCCTGGTTCA		R- ATGACTTCCAAGCTGGCCGTGGCT
**ADAM9**	F- TTAGTGAAGATAGTGGATTGAGTACAGCTT	**IL10**	F- ATGCAGGACTTTAAGGGTTACTTGGGTT
	R- TGTTGGAGCCATGACATGCT		R- ATTTCGGAGAGAGGTACAAACGAGGTTT
**ADAM10**	F- TTTGGATCCCCACATGATTCTG	**IL12**	F- TCGCGTTCACAAGCTCAAGT
	R- GGTTGGCCAGATTCAACAAAAC		R- CAAACCTGACCCACCCAAGA
**ADAM12**	F- GGAATTGTCATGGACCATTCAG	**IL17**	F- GTCTGGGCGCAGGTATGTGG
	R- TTCCTGCTGCAACTGCTGAACA		R- CACCGTGGAGACCCTGGAGGC
**ADAM15**	F- AACATGGACCACTCCACCAGCA	**IL18**	F- CAGACAACTTTGGCCGACTTCA
	R- TTCGAAGAGGCAGCTGCCCATT		R- ACACAAACCCTCCCCACCTAACT
**ADAM17**	F- TACAAAGGAAGCTGACCTGGTT	**IRAK4**	F- CAGACTCTCTTGCTTGGATGGT
	R- TTCATCCACCCTCGAGTTCCCA		R- AGCTGACCCTGAGCAATCTT
**ADAM23**	F- TAGGGATCCCAAAGCTATTTGAGCCCA	**IRF3**	F- GTTCTGTGTGGGGGAGTCAT
	R- ATGAAGATTCGGTGGGCA		R- CTGTTGGAAATGTGCAGGTC
**ADAM33**	F- TGGTTCAAGTTTCGGTGCCGAG	**MMP1**	F- CAGTGGTGATGTTCAGCTAGCTCA
	R- GAGTGGCCTGATCACCCTCA		R- GCCGATGGGCTGGACA
**ADAMTS1**	F- CAGCCCAAGGTTGTAGATGGTA	**MMP2**	F- GAGGACTACGACCGCGACAA
	R- TTCACTTCGATGTTGGTGGCTC		R- CTTCACTTTCCTGGGCAACAA
**ADAMTS2**	F- GAACCATGAGGACGGCTTCTCCT	**MMP3**	F- GCCAGGGATTAATGGAGATG
	R- GGCTGCAGCGGGACCAGTGGAA		R- ATTTCATGAGCAGCAACGAG
**ADAMTS4**	F- GCAACGTCAAGGCTCCTCTT	**MMP7**	F- GTGGGAACAGGCTCAGGACTATCTCAA
	R- CTCCACAAATCTACTCAGTGAAGCA		R- CACATTGGGCTTCTGCATTATTACTA
**ADAMTS5**	F- AGGAGCACTACGATGCAGCTATC	**MMP9**	F- CCTGGAGACCTGAGAACCAATC
	R- CCCAGGGTGTCACATGAATG		R- GATTTCGACTCTCCACGCATCT
**ADAMTS15**	F- GCCTGGCAGAAGAAGCTGAAC	**MMP11**	F- GAGCAGGTGCGGCAGACGA
	R- GCTGTCCAGGAAGTCGGTGAT		R- CGAAAGGTGTAGAAGGCGGACA
**ANGPT1**	F- TTCTCTTCCCAGAAACTTCA	**MMP13**	F- ATCCCTTGATGCCATTACCA
	R- ACTGAACCTGACCGTACACATCTCCGACTT		R- AAGAGCTCAGCCTCAACCTG
**ANGPT2**	F- TATGGAAAACAACACTCAG	**MMP14**	F- GCCCAATGGGAAGACCTACT
	R- ACTGAACCTGACCGTACATTCTGTACTGCATTCTGCTG		R- AGGGTACTCGCTGTCCACTG
**ANXA2**	F- CTCTACACCCCCAAGTGCAT	**MyD88**	F- TGGCACCTGTGTCTGGTCTA
	R- TCAGTGCTGATGCAAGTTCC		R- ACATTCCTTGCTCTGCAGGT
**CCL3**	F- CTTGCTGTCCTCCTCTGCAC	**NFκB**	F- TCTCCCTGGTCACCAAGGAC
	R- TCACTGGGGTCAGCACAGAC		R- TCATAGAAGCCATCCCGGC
**CLDN3**	F- CTGCTCTGCTGCTCGTGTCC	**TEK**	F- TGTTCCTGTGCCACAGGCTG
	R- TTAGACGTAGTCCTTGCGGTCGTAG		R- CACTGTCCCATCCGGCTTCA
**COX2**	F- ATCATAAGCAGGGCCAGCT	**TGFβ1**	F- GCAACAATTCCTGGCGATAC
	R- AAGGCGCAGTTTACGCTGTC		R- AAGGCGAAAGCCCTCAAT
**CXCL16**	F- TCTCAAAGAATGTGGACATGC	**TIMP-1**	F- CCGCAGCGAGGAGTTTCTC
	R- CAGGGGTGTGGATATCTGAA		R- GAGCTAAGCTCAGGCTGTTCCA
**CXCR4**	F- AGCTGTTGGCTGAAAAGCTGGTCTATG	**TIMP-2**	F- CGACATTTATGGCAACCCTATCA
	R- GCGCTTCTGGTGGCCCTTGGAGTGTG		R- GCCGTGTAGATAAACTCTATATCC
**EGF**	F- ACATCAAATATCCTCAATGG	**TIMP-3**	F- GCGTCGGAGGTTAAGGTTGTT
	R- GTGGCATCAAGACCGGGCTGC		R- CTCTCCAAAATTACCGTACGCG
**FGF-2**	F- ACCCCGACGGCCGA	**TLR2**	F- CAGGGCTCACAGAAGCTGTAA
	R- TCTTCTGCCTTGAAGTTGTAGCTTGA		R- GCCCAGGGAAGAAAAAGAATC
**FN**	F- CCACAGTCGGGTCAGGAG	**TLR3**	F- TAGCAGTCATCCAACAGAATCAT
	R- CTGGCCGAAAATACATTGTAAA		R- AATCTTCTGAGTTGATTATGGGTAA
**ICAM1**	F- AGGCCACCCCAGAGGACAAC	**TLR4**	F- ACTCCCTCCAGGTTCTTGATTAC
	R- CCCATTATGACTGCGGCTGCTA		R- CGGGAATAAAGTCTCTGTAGTGA
**IFNα**	F- TGGCTGTGAAGAAATACTTCCG	**TLR5**	F- GATTCTTGCCCACCACAT
	R- TGTTTTCATGTTGGACCAGATG		R- GGTTCGCTGTAAGGTTGAT
**IFNβ**	F- TCTCCACGACAGCTCTTTCCA	**TLR7**	F- CGGCTTGATTTACTCTCCAT
	R- ACACTGACAATTGCTGCTTCTTTG		R- CAGTGGTCAGTTGGTTGTGG
**IGF-1**	F- TTGTGATTTCTTGAAGGTGAAGATG	**TLR9**	F- CTTCCCTGTAGCTGCTGTGTCC
	R- CGTGGCAGAGCTGGTGAAG		R- CCTGCACCAGGAGAGACAG
**IGFBP-2**	F- GCAGGTTGCAGACAATGGCG	**TNF**	F- CCAGGGACCTCTCTCTAATCAGC
	R- GTGGTCCAGCTTCTTGGGC		R- CTCAGCTTGAGGGTTTGCTACAA
**IL1**	F- TAGTAGCAACCAACGGGAAG	**VEGFA**	F- GCAGAATCATCACGAAGTGG
	R- CTCTGAGTCATTGGCGATG		R- GCAACGCGAGTCTGTGTTTTTG
**IL4**	F- CCACGGACACAAGTGCGATAT	**VEGFC**	F- GCCACGGCTTATGCAAGCAAAGAT
	R- CGTAACAGACATCTTTGCTGCC		R- AGTTGAGGTTGGCCTGTTCTCTGT
**IL5**	F- AAAGGCAAACGCAGAACGTGT	**VEGFD**	F- CGATGTGGTGGCTGTTGCAATGAA
	R- CTCTTGGAGGTGCCTAGTGT		R- GCTGTTGGCAAGCAAGCACTTGACAACCT
**IL6**	F- GAACTCCTTCTCCACAAGCGCCTT		
	R- CAAAAGACCAGRGATGATTTTCACCAGG		

F: forward primer; R: reverse primer.

## Results

### MMP-11 Immunostaining

**Figure 1:**
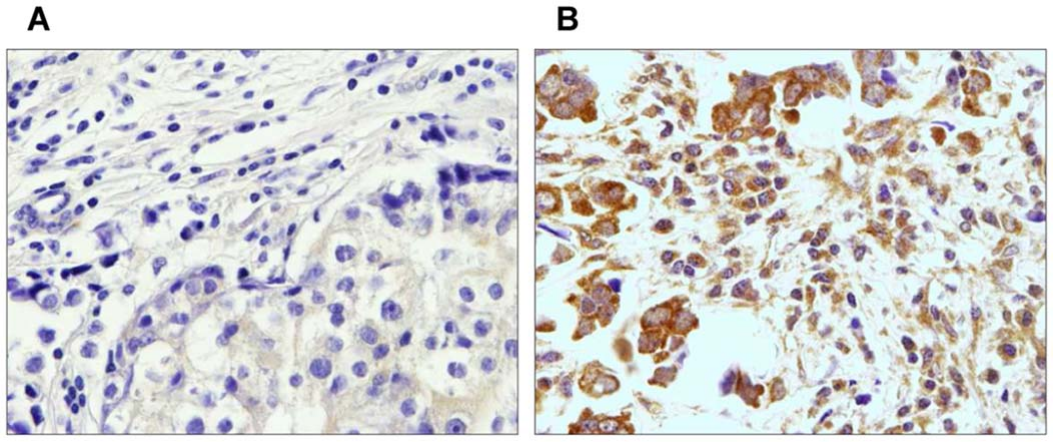
Representative examples of MMP-11 immunostaining in MICs (x400). (a) MMP-11 negative staining in MICs. (b) MMP-11 positive staining in MICs. The red arrow represents tumor cells and the green arrow represents MICs.

We analyzed MMP-11 expression by MICs in breast carcinomas samples, to distinguish the two principal groups of tumors (Figure 1). Immunostaining for this protein has a cytoplasmic location in all positive cases. It was very easy to distinguish “positive” from “negative” cases, because all MMP-11 positive cases showed at least as 70% positive MICs; whereas in MMP-11 negative cases, no more than 10% of MICs were stained. We did not find cases with MMP-11 positive MICs and negative tumor cells; however, we found cases with MMP-11 positive tumor cells and negative or positive expression by MICs. T-cells (CD3^+^), B-cells (CD20^+^) and macrophages (CD68^+^) counts showed no significant differences between each group of tumors studied (data not shown).

### Preliminary Screening of Factors Related to MMP11-expression

**Figure 2:**
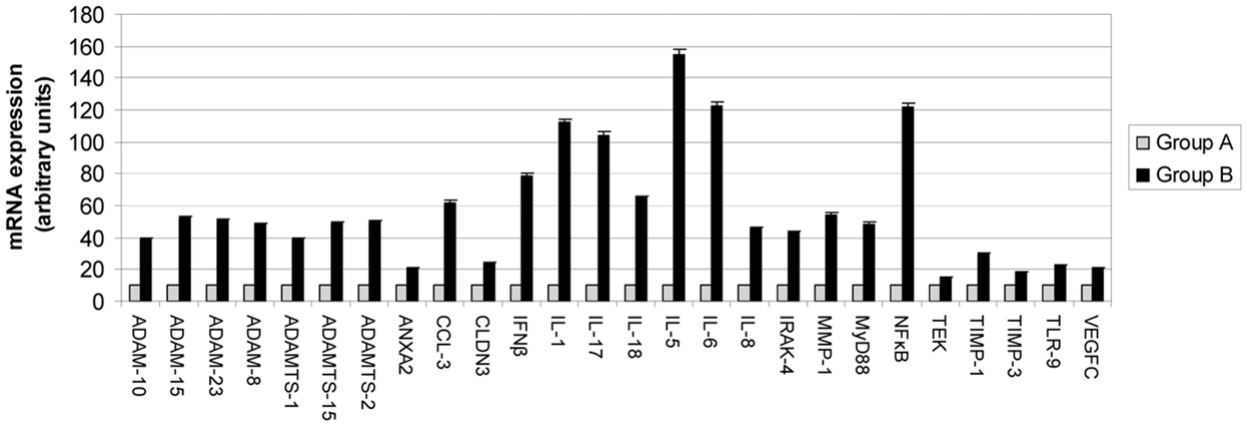
Real-time PCR analysis of factors differentially expressed between the two main tumor groups. Group A: tumors with MMP-11 negative MICs. Group B: tumors with MMP-11 positive MICs. Data represent the mean ± SD of three independent experiments.

In an initial approach, we analyzed a sample size of 6 patients whose tumors have MMP-11 negative MICs (Group A) and 6 patients whose tumors have MMP-11 positive MICs (Group B). In these groups we analyzed by real-time PCR the differential expression of 65 factors related with tumor progression and inflammation (Table 1 and 3) and found differences in the RNA expression of 26 factors (Figure 2), that were therefore related with MMP-11 expression by MICs. All factors show raised levels in tumors with MMP-11 positive MICs. However, carcinoma samples with MMP-11 positive MICs showed a more important increase in the mRNA level of 19 factors: IL-1, −5, −6, −8, −17 and −18, ADAM-8, −10, −15, and −23, ADAMTS-1, −2, and −15, IFNβ, MMP-1, as well as mediators related to inflammation (CCL-3, IRAK-4, MyD88 and NFκB). It was remarkable that expression of several factors such as IL-1, −5, −6 and −17, NFκB and IFNβ was increased at least 8 to 15 fold in Group A compared with Group B samples. Initially, we considered factors increased at least 10 fold but with this threshold we discarded an important cytokine as IFNβ, therefore, we decided to include IFNβ and thus decrease the threshold to 8 fold. Thus, these six factors more differentially expressed between both groups of tumors are the factors selected to study differences between groups later on.

### Relationship Between IL-1, −5, −6 and −17, IFNβ and NFκB, and Distant Metastasis Development

**Figure 3:**
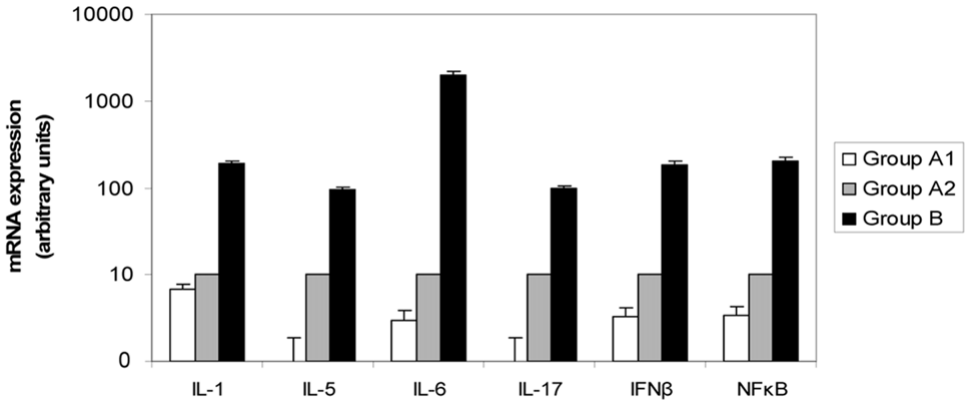
Real-time PCR analysis of the 6 most differentially expressed factors between the three tumors groups. Group A1: tumors with MMP-11 negative MICs and without distant metastasis. Group A2: tumors with MMP-11 negative MICs and distant metastasis. Group B: tumors with MMP-11 positive MICs and distant metastasis. Data represent the mean ± SD of three independent experiments.

We analyzed by real-time PCR the expression of the selected factors (IL-1, −5, −6 and −17, IFNβ and NFκB) more differentially expressed in the preliminary screening, in a wider tumor population consisting of three differentiated groups according to MMP-11 expression by MICs and to distant metastasis development (n = 15 in each group) (Figure 3). The results indicate that the expression levels of these inflammatory factors were significantly higher in tumors with MMP-11 positive MICs and that develop distant metastasis during the follow-up period (Group B1), compared with tumors with MMP-11 negative MICs and distant metastasis (Group A2), or compared with tumors with MMP-11 negative MICs and without distant metastasis (Group A1), which showed the lower levels of these factors.

Thus, these results contribute to identify a tumor group with up-regulated inflammatory-related genes, which present worse prognosis. The classification of these tumor populations in good or bad prognosis was based on the expression of MMP11 by MICs, as described previously by our group [3]. Our study emphasizes the importance of IL-1, −5, −6 and −17, IFNβ and NFkB in promoting distant metastasis and recurrence, as demonstrated by their high expression in tumors from patients with a higher rate of distant metastasis development (97.6%) [3]. Some of these molecules implicated in the cross-talk between the tumor and the inflammatory microenvironment may emerge as attractive targets in breast cancer.

### Expression of IL-1, −5, −6 and −17, IFNβ and NFκB by Tumor and Stromal Cells

We analyzed by immunohistochemistry the expression of IL-1, −5, −6 and −17, IFNβ and NFκB in three differentiated groups according to MMP-11 expression by MICs and to distant metastasis development (n = 15 in each group). Table 4 indicates that all cell type contribute to the overall expression of these factors, except NFκB which was expressed only by tumor cells. In addition, IL-6 was differentially expressed by fibroblasts between each group (p = 0.006), showing fewer positive cases in Group A2. In the same way, Group A2 show less positive cases for IL-17 by tumor cell (p = 0.008). In addition, IFNβ expression by tumor cells was similar in Group A1 and B1. Generally, differential expression of these factors is not dependent on one cell type.

**Table 4:** Percentage of cases positive for IL-1, −5, −6, −17, IFNβ and NFκB by each cell type as function of group.

	Group A1			Group A2			Group B1		
	Tumor cell	MIC	Fibroblast	Tumor cell	MIC	Fibroblast	Tumor cell	MIC	Fibroblast
**IL-1**	90.9	90.9	90.9	100	100	100	100	90	100
**IL-5**	95	50	25	89	58	22	100	62.5	12.5
**IL-6**	89.5	52.6	36.8 *	66.7	66.7	66.7	100	88.9	100
**IL-17**	100 *	78.9	89.5	66.7	66.7	66.7	100	100	88.9
**IFNβ**	90.9 *	100	100	33.3	100	100	90	90	100
**NFκB**	81.8	0	0	66.7	0	0	80	0	0

MIC: mononuclear inflammatory cells; *p<0.05.

## Discussion

The present study consists in a molecular characterization of the inflammatory process implicated in the tumor progression of breast cancer. In this study we used a classification of tumors in good or bad prognosis based on the expression of MMP11 by MICs, as described previously by our group [3]. The accomplishment of a pilot study has allowed us to perform a preliminary screening of molecules associated with tumor progression and inflammation, and to analyze the relationship between specific inflammatory factors and distant metastasis development in breast carcinomas based on MMP-11 expression by MICs.

Inflammatory cells can represent up to 50% of the total tumor mass in an invasive mammary carcinoma, and include macrophages, plasma cells, mast cells, T and B-lymphocytes [14], [15]. Historically, the infiltrating tumor-associated leukocytes have been considered as an intrinsic mechanism of defense against tumor development [15], [16]. Our results agree with increasing evidences indicating that leukocyte infiltration can promote changes leading to a more aggressive tumor phenotype in the scope of angiogenesis, tumor growth, invasion and metastasis [14], [17]. Inflammatory cells probably influence tumor progression by secreting factors like cytokines, growth factors, chemokines and proteases, which stimulate the proliferation and invasiveness of tumor cells. According to this, recently Tan et al. have characterized a type of tumor-infiltrating lymphocytes that stimulate the development of breast cancer metastasis through signals related to the transcription factor NFκB [18].

Nevertheless, the prognostic significance of the lymphoid infiltrate at the tumor site remains controversial perhaps because the evaluation criteria for these tumor infiltrates are not sufficiently standardized to yield reliable and reproducible results in different institutions. Therefore, our results may contribute to a better characterization of the inflammatory phenotype of mammary carcinomas associated with unfavorable prognosis. Altogether, these results suggest that identification of these cases turns out to be an important key in the molecular biology of mammary carcinomas associated with tumor progression, and depending not only on the tumor cells themselves but also on the surrounding inflammatory cell infiltrate.

Our results demonstrate that, of the 65 factors analyzed and related to the inflammatory process and tumor progression, those related to MMP-11 expression by MICs in the intratumoral stroma were IL-1, −5, −6, −8, −17, −18, MMP-1, TIMP-1, ADAM-8, −10, −15, −23, ADAMTS-1, −2, −15, CCL-3, Annexin A2, IFNβ, Claudin3, IRAK-4, MyD88 and NFκB. Of them, factors more differentially expressed between both main types of tumors were IL-1, −5, −6, −17, IFNβ and NFκB. These latter factors were analyzed in a wider tumor sample, in which we confirmed the higher expression of those factors in tumors infiltrated by MMP-11 positive MICs and that the expression not depend of only one cell type. Thus, our study contributes to a better biological characterization of mammary carcinomas, especially with regard to the molecular profile of its inflammatory component.

These factors showing an increased expression level in tumors infiltrated by MMP-11 positive MICs, like IL-1, −5, −6, −17, IFNβ and NFκB, have a great biological interest because of their relation with tumor progression. IL-1 is essentially produced by activated macrophages, and induces a great variety of genes like IL-5, IL-6, oncogenes (c-fos, c-myc, c-jun), IFN-β and collagenases. Different experimental models have shown that local production of IL-1 influences tumor growth and metastasis development, either through direct proliferative effects or through the activation of the inflammation and angiogenesis signaling [19], [20]. The production of IL-1 by tumor or stromal cells has been associated with an aggressive tumor phenotype in several types of mouse and human cancers [21]. These data support our results, in which patients with a higher frequency of metastasis (97.6%) present a higher expression of IL-1.

IL-5 is essentially produced by T-helper type-2 lymphocytes and mast cells, stimulates B cells growth and increases the production of immunoglobulins. This interleukin has not yet been described as an important factor in the development of breast cancer metastasis, but our results indicate that could be an important target to analyze in these cases.

IL-6 seems to play an important role in the resistance to the apoptotic process. IL-6 is produced by stromal cells like T-cells, fibroblasts or monocytes and also by tumor cells. Some studies show the role of IL-6 in tumor cells growth *in vitro*, but its exact role is still unclear. Also, studies evaluating IL-6 expression in mammary carcinomas, show contradictory results. Marrogi et al. analyzed the expression profile of IL-6 in 19 mammary carcinomas and detected no mRNA expression [22]. Nevertheless, other studies have detected and quantified IL6 expression in breast cancer [23], [24]. Bachelot et al. studied the clinical meaning of vascular endothelial growth factor (VEGF) and IL-6 expression in hormone-refractory mammary carcinomas and observed that presence of IL-6 in patient’s serum (but not VEGF), was correlated with a shorter survival [25]. In our case, we found that intratumor expression of IL-6 correlates with a higher risk of metastasis.

In the last few years, IL-17 has been considered as a key link between adaptive and innate immunity, and also plays a critical role in inflammation and autoimmune diseases. In spite of the role of IL-17 in autoimmunity, it is relatively little known about its function in cancer, and the published data are still contradictory. Some studies support its role in tumor progression, probably due to the stimulation of angiogenic factors [26], [27]. On the contrary, other studies suggest that IL-17 promotes tumor rejection through a T-cell-dependent mechanism [28]. CD8^+^ T-cells and non-T-cells have been reported to produce Th17 cytokines [29], including IL-17, but the role of non-T-cell-derived IL-17 remains to be further defined. Our data suggest that IL-17 contributes to tumor progression and aggressiveness, showing an expression decrease in at least 100 fold in tumors that do not develop metastasis compared to tumors with unfavorable prognosis.

The production of IFNβ by T and B-cells, macrophages, fibroblasts, or endothelial cells among others, is induced by other cytokines like IL-1, IL-2, TNF and CSF. The function of IFNβ in breast cancer progression has already not been described. Nevertheless, this cytokine well-known because of its role in antiviral immunity, can be related with the recent association between human papilloma virus and breast cancer [30], [31], [32].

Nuclear factor kappa (NFkB) has a specific role in tumor progression, and also has been associated to cancer stem cells survival [33]. NFkB regulates the expression of numerous antiapoptotic proteins associated with tumor survival (bcl-xl, bcl-2, XIAP, c-FLIP, IAP-1, IAP-2, and survivin), as well as genes associated with tumor progression (cyclin D1, c-myc and COX-2). In addition, numerous data support the role of NFkB in the regulation of tumor inflammation and progression [34].

The result of the present study was that tumors developing worse prognosis and identified by MMP-11 expression by intratumoral MICs, showed an up-regulation of inflammatory-related genes. The classification of these tumor groups in good or bad prognosis was based on the expression of MMP-11 by MICs, as described previously by our group [3]. Our study emphasizes the importance of IL-1, −5, −6 and −17, IFNβ and NFkB in promoting disease metastasis and recurrence, as demonstrated by their high expression in tumors from patients with a higher rate of distant metastasis development (97.6%) [3]. Some of these molecules implicated in the cross-talk between the tumor and inflammatory microenvironment may emerge as attractive targets in breast cancer. Therefore, these data contribute to a better biological characterization of tumors and open up the possibility of undergoing new studies to determine which cell type specifically express those factors, and their biological signification.
